# Serial Analysis of Gene Expression in *Plasmodium berghei *salivary gland sporozoites

**DOI:** 10.1186/1471-2164-8-466

**Published:** 2007-12-19

**Authors:** Isabelle Rosinski-Chupin, Thomas Chertemps, Bertrand Boisson, Sylvie Perrot, Emmanuel Bischoff, Jérôme Briolay, Pierre Couble, Robert Ménard, Paul Brey, Patricia Baldacci

**Affiliations:** 1 Biochimie et Biologie Moléculaire des Insectes, Institut Pasteur, 28 rue du Dr Roux, 75724, Paris cedex 15, France; 2 Biologie et Génétique du Paludisme, Institut Pasteur, 28 rue du Dr Roux, 75724, Paris cedex 15, France; 3 Plateforme 2: Puces à ADN, Institut Pasteur, 28 rue du Dr Roux, 75724, Paris cedex 15, France; 4 DTAMB, Université de Lyon; IFR 41; 43 boulevard du 11 Novembre 1918, 69622 Villeurbanne cedex, France; 5 Centre de Génétique Moléculaire et Cellulaire, UMR 5534, C.N.R.S.-Université de Lyon, 69622 Villeurbanne cedex, France

## Abstract

**Background:**

The invasion of *Anopheles *salivary glands by *Plasmodium *sporozoites is an essential step for transmission of the parasite to the vertebrate host. Salivary gland sporozoites undergo a developmental programme to express genes required for their journey from the site of the mosquito bite to the liver and subsequent invasion of, and development within, hepatocytes. A Serial Analysis of Gene Expression was performed on *Anopheles gambiae *salivary glands infected or not with *Plasmodium berghei *and we report here the analysis of the *Plasmodium *sporozoite transcriptome.

**Results:**

Annotation of 530 tag sequences homologous to *Plasmodium berghei *genomic sequences identified 123 genes expressed in salivary gland sporozoites and these genes were classified according to their transcript abundance. A subset of these genes was further studied by quantitative PCR to determine their expression profiles. This revealed that sporozoites modulate their RNA amounts not only between the midgut and salivary glands, but also during their storage within the latter. Among the 123 genes, the expression of 66 is described for the first time in sporozoites of rodent *Plasmodium *species.

**Conclusion:**

These novel sporozoite expressed genes, especially those expressed at high levels in salivary gland sporozoites, are likely to play a role in *Plasmodium *infectivity in the mammalian host.

## Background

The sporozoite is the stage of the malaria parasite transmitted from the mosquito vector to the mammalian host. The success of infection depends on the sporozoites leaving their site of inoculation in the dermis, rapidly attaining the liver, invading hepatocytes, and developing therein [1-4]. This results in the release of thousands of merozoites from the infected hepatocytes that subsequently invade red blood cells, causing the malaria disease [5]. Sporozoites are formed within oocysts of the mosquito midgut and are initially poorly infectious. They migrate to mosquito salivary glands (SG) and must undergo a developmental programme, with concomitant changes in gene expression, in order to become highly infectious to the mammalian host. These SG sporozoites exhibit a circular gliding movement and in certain conditions can elicit a strong protective immune response in the mammalian host [6-8].

Recent technological advances have improved our understanding, at the molecular level, of the *Plasmodium *parasite including the less well known sporozoite stage. The completion of the *Plasmodium falciparum *(*P. falciparum*) genome sequence allowed the analysis of gene expression at different stages of the parasite life cycle with microarrays [9]. The data showed that approximately 2000 genes are expressed in SG sporozoites, 500 of which are expressed at high levels and over a 100 of these are not expressed at significant levels in blood stages. In addition, proteomic analyses detected 274 proteins in *P. falciparum *sporozoites [10,11].

*Plasmodium *species infecting rodents are powerful laboratory models as they are more easily amenable to genetic studies and their genomes have also been sequenced [11,12]. However, the transcriptome of sporozoites from these species is less well known and has been obtained mainly from *Plasmodium yoelii *(*P. yoelii*) cDNA libraries [13] and Suppressive Subtractive Hybridisation studies [14,15]. In addition, 119 proteins have been identified in *Plasmodium berghei *(*P. berghei*) sporozoites, of which 34 are sporozoite specific (PlasmoDB release 5.1).

We recently reported a SAGE aimed at characterising *Anopheles gambiae *(*A. gambiae*) SG genes that are differentially expressed in response to infection with *P. berghei *sporozoites [16]. In that study 530 tag sequences, found exclusively in libraries from infected mosquitoes, were identical to *P. berghei *genomic sequences, and 41 of these were readily annotated. Considering the data presently available for the transcriptome of *P. berghei *sporozoites, we decided that it would be worthwhile to annotate the remaining tags. SAGE allows gene-expression-profiling based on the quantification of short 14–15 nucleotide (nt) sequence tags, each sequence being, in theory, associated with the transcript of a single gene. It provides an overall estimation of the abundance of transcripts, while requiring no *a priori *information about the sequence of the transcripts to be studied [17,18]. However, only short cDNA sequences, usually located in the 3'UTR, are obtained and consequently the attribution of a tag sequence to a gene is highly dependent on the quality of annotation of the genome of the organism studied and available cDNA or EST studies [19,20]. On the other hand, positioning tags on the genomic sequence can provide information on both the orientation of a transcript and the length of the 3' UTR.

The tags from our SAGE were annotated by combining information on their position in the *P. berghei *genomic sequences, predicted gene models and ESTs from *P. berghei *and *P. yoelii*. We unambiguously identified 123 genes expressed in *P. berghei *sporozoites, of which 66 are detected for the first time in rodent *Plasmodium *species. The hierarchical classification of these transcripts according to the abundance of the tags was confirmed by qPCR and the characterisation of the gene structure and/or gene expression was undertaken for some. Finally, our results provide evidence that mRNA levels may vary not only between midgut and SG sporozoites but also during storage of the sporozoites in the SG.

## Results

### Annotation of *Plasmodium berghei *tags: rationale

**Figure 1:**
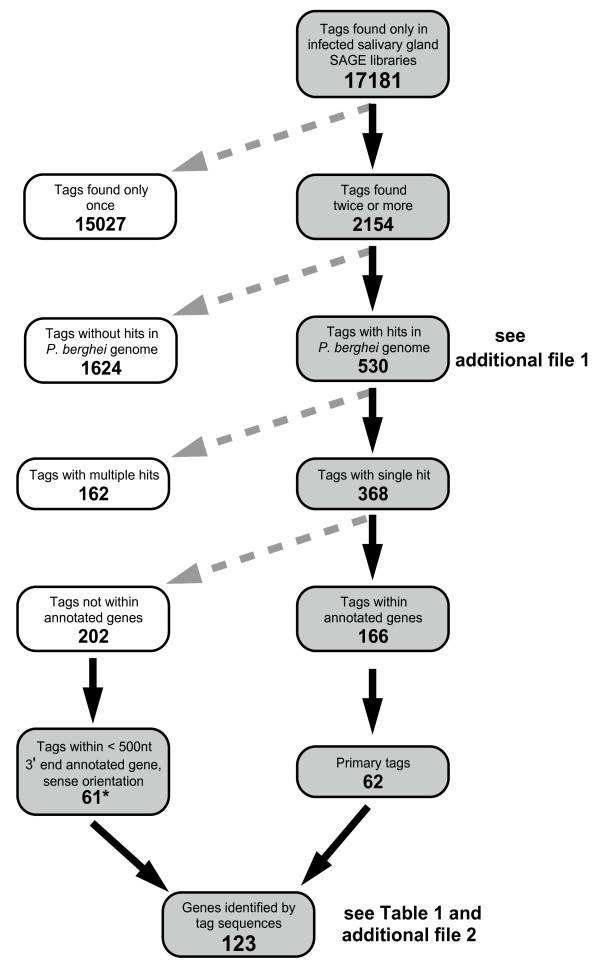
Flow chart of the rationale used to select Tag sequences from the SAGE library. Shaded boxes correspond to tag sequences retained, clear boxes correspond to tag sequences rejected.

SAGE libraries were constructed from four different SG RNA preparations: SG isolated from *A. gambiae *mosquitoes 14, or 18, days after a blood meal on *P. berghei *infected mice and SG isolated 14, or 15–18, days after feeding on uninfected mice [16]. Sequence analysis showed that among the tags identified in the infected SG libraries, 17181 were absent from uninfected SG libraries and were thus considered as potential *P. berghei *sequences (see Figure 1). These sequences were then screened using the following criteria: 1) to be found more than once in the cumulative libraries, 2) to give a single hit in the *P. berghei *genome, 3) to be derived from the most 3' *Nla*III site predicted in the annotated gene sequence in the sense orientation ("primary" tags in Figure 1) or to be found within 500 nt of the stop codon of an annotated gene in the sense orientation.

We found 2154 tag sequences at least twice in the cumulative libraries and 530 of these gave one or more perfect matches by BLASTN comparison on *P. berghei *genomic sequences and annotated genes. These tag sequences and their annotations are provided in Additional file 1. We discarded from further analysis 162 tags that gave multiple hits in the genome due to the impossibility of assigning them to a unique gene. Of the 368 tags that gave a single hit in the genome, 166 were found within annotated genes and 62 of these were derived from the most 3' *Nla*III predicted site.

Sampling of the 3' untranslated regions of about 30 *Plasmodium *genes showed that 77% of them were 500 nt in length (I.C. unpublished observation). Therefore, we analysed in more detail the position in the genome of the 202 tags that were not found within annotated genes. Sixty-three tag sequences, found within 500 nt downstream of the stop codon of an annotated gene and in the sense orientation, were retained in the analysis as it seemed likely that they came from the 3' end of the adjacent genes. As two genes were identified with two different tag sequences respectively, only one tag was retained for each gene, resulting in a total of 61 (see Figure 1).

In total, 123 identifying tag sequences fulfilled all our criteria (see Table 1 and Additional file 2). BLASTN analysis of the 123 genes identified by these tags showed that 55 aligned with known transcripts from *P. yoelii *or *P. berghei *sporozoites, whereas the remaining 68 did not. Among the latter, proteomic approaches have detected PB000876.00.0 in sporozoites and a study by Ishino *et al.*[21] showed that PB000892.00.0 (*Pbs36*) is also present. It should be noted that the criteria used have resulted in the elimination of the majority of tag sequences, including several tags corresponding to genes known to be expressed in SG sporozoites (see Additional file 1). For example, two tags aligned with PB001026.00.0, coding for circumsporozoite protein (CS). The most abundant tag, found 77 times, gave 4 hits in the genome and the second tag sequence was in the antisense orientation. Although it is likely that the most abundant tag originated from the *CS *transcript, it was not taken into account in order to be consistent in the analysis. Another example concerned two tags aligning with PB000892.03.0 (*UIS3*), a gene known to be upregulated in sporozoites [14]. Again the most abundant tag gave multiple hits in the genome and the second was in the antisense orientation.

Taken together this SAGE analysis has identified 66 novel sporozoite expressed genes (indicated as *SIS *on Table 1).

**Table 1:** List of 123 genes identified by SAGE in *P. berghei *salivary gland sporozoites

		**times found**							
							
	**Tag ID**	**d14**	**d18**	**total**	**GeneDB ID**	**Known as**	**Sporozoite expression**	**Pf orthologue**	**Predicted Pb/Pf protein domain and/or function**
**group 1**	tag 335	60	39	99	PB402615.00.0*		d	Pfgenefinder_63r	
	tag 183	6	89	95	PB100551.00.0	*UIS4*	a, d	--	SP, TM
	tag 190	9	59	68	PB107461.00.0	*UIS7*	d	--	TM
	tag 269	6	25	31	PB000374.03.0	*TRAP*	d, 1, P	PF13_0201	SP, vWA-like, TSP1
	tag 226	11	13	24	PB001063.00.0	*S23*	d, 1	PF08_0088	Pb-reticulocyte binding, SP, TM

**group 2**	tag 435	10	7	17	PB000252.01.0	*S13, SPECT2*	d, Pb, 1, P	PFD0430c	SP, MAC/Perforin domain
	tag 168	4	12	16	PB000317.00.0*		d, 1, P	PF08_0008	SP, TM, GPI
	tag 146	8	8	16	PB000881.01.0	*S21/PbTRSP*	d, 1, P	PFA0200w	TM, TSP1
	tag 112	11	2	13	PB000649.01.0	*ECP1*	Δ, 1, P	PFB0325c	Papain family cysteine protease
	tag 110	2	11	13	PB104017.00.0	*S12*	d	--	SP
	tag 200	2	9	11	PB000400.01.0*	*SIS1*	Δ, 1	PFE0395c	Pf38, Sexual stage antigen, SP, TM, GPI
	tag 75	3	8	11	PB000484.00.0	*UIS10/PbPL*	a	PFF1420w	Phosphatidylcholine-sterol acyltransferase
	tag 50	4	6	10	PB000891.00.0	*Pbs36p*	d, 1	PFD0215c	Pf52, Sexual stage antigen, SP

**group 3**	tag 76	1	8	9	PB104912.00.0*		d, 1	PF11_0283	SP, TM
	tag 264	3	5	8	AB125370	*SPECT*	d, 1, P	MAL13P1.212	SP
	tag 278	4	4	8	PB000757.02.0	*SIS2*	Δ, 1, P	PF14_0425	Fructose-1, 6-bisphosphate aldolase
	tag 319	1	7	8	PB108190.00.0*	*UIS1*	a	--	Serine/Threonine protein kinases, 9 TM
	tag 136	2	5	7	PB100963.00.0		d	--	Guanylyl cyclase, TM
	tag 245	0	6	6	PB000780.01.0*		d, 1	PFI1230c	4 TM, asparagine-rich
	tag 530	0	5	5	PB000264.00.0*		d	PF14_0044	SP
	tag 118	0	5	5	PB000411.03.0*	*SIS3*	Δ	--	
	tag 294	1	4	5	PB000464.00.0	*S25*	d, 1, P	PFL0545w	Kinesin-related protein
	tag 280	0	5	5	PB000817.02.0		d, 1, P	PF08_0054	Heat shock 70 kDa proteinn
	tag 268	1	4	5	PB001070.03.0		d	PFF0685c	Fe-only hydrogenase
	tag 193	1	4	5	PB101172.00.0		d	--	
	tag 92	2	2	4	PB000161.03.0		d, 1	PFI0955w	sugar transporter, 6TM
	tag 201	0	4	4	PB000787.03.0	*SIS4*	Δ, 1	PFI0320w	Arginase family
	tag 101	0	4	4	PB105090.00.0	*SIS5*	Δ	--	TM
	tag 233	1	3	4	PB106293.00.0*		d	MAL13P1.365	SNARE
	tag188	2	2	4	PB107843.00.0*	*SIS6*	Δ, 1	PF10_0290	TM
	tag 62	3	1	4	PB108391.00.0		d, 1, P	PF08_0072	TM
	tag 276	1	3	4	PB000632.00.0	*SIS7*	Δ, 1	PF14_0518	nifU protein, putative
	tag 354	3	0	3	PB000175.03.0*	*SIS8*	Δ, 1	PFD0915w	TM
	tag 171	1	2	3	PB000416.02.0		d, 1	PFD0825c	RNA-binding protein of pumilio/mpt5 family
	tag 326	2	1	3	PB000422.00.0		d, 1, P	PF07_0126	
	tag 213	0	3	3	PB000468.02.0	*SIS9*	Δ, 1	PFL0335c	Eukaryotic translation initiation factor 5
	tag 197	0	3	3	PB000626.00.0		d, 1	PF14_0224	PP1-like protein serine/threonine phosphatase
	tag 68	1	2	3	PB000674.01.0	*SIS10*	Δ, 1, P	PF13_0058	RNA recognition motif
	tag 170	0	3	3	PB000876.00.0		Pb, 1, P	MAL8P1.78	Small heat shock protein
	tag 236	0	3	3	PB000892.00.0	*Pbs 36*	Δ, 1	PFD0210c	Pbs36, Sexual stage antigen, PS
	tag 20	0	3	3	PB000911.00.0*	*SIS11*	Δ, 1	PFE0965c	Vacuolar ATP synthetase, 4 TM
	tag 8	1	2	3	PB001101.02.0	*SIS12*	ΔΔ	PF14_0395	
	tag 119	0	3	3	PB001143.02.0	*SIS13*	Δ, 1, P	PF14_0083	Ribosomal protein S8e
	tag 122	1	2	3	PB001247.02.0	*S14*	d, 1, P	PFL0370w	
	tag 127	0	3	3	PB001432.02.0	*SIS14*	Δ, 1	PFE0340c	Peptidase S54, rhomboid, 7 TM
	tag 155	3	0	3	PB001575.02.0	*SIS15*	Δ, 1	PFB0270w	SP, TM, Fe-S metabolism associated domain
	tag 150	0	3	3	PB104165.00.0		d, 1	PFI0675w	
	tag 305	0	3	3	PB104461.00.0		d	--	SP, TM
	tag 350	0	3	3	PB105607.00.0		d	--	2 TM
	tag 106	1	2	3	PB105732.00.0		d	--	TM
	tag 196	0	3	3	PB106033.00.0	*SIS16*	Δ, 1	PF10_0296	
	tag 41	1	2	3	PB107193.00.0*	*SIS17*	ΔΔ	PFF1195c	
	tag 121	1	2	3	PB108098.00.0		d	--	TM
	tag 157	2	1	3	PB300784.00.0		d, 1	MAL8P1.154	
	tag 362	0	2	2	PY01277**	*SIS18*	ΔΔ	PFL2080c	
	tag 142	0	2	2	PB000006.00.0	*SIS19*	Δ, 1	PFE0995c	
	tag 265	0	2	2	PB000064.03.0	*SIS20*	ΔΔ	PFB0510w	3 TM
	tag 143	0	2	2	PB000092.02.0	*UIS2*	a, 1, P	PF14_0614	
	tag 355	1	1	2	PB000094.03.0	*SIS21*	Δ, 1, P	MAL12P1.180	Arginyl-tRNA synthetase
	tag 15	1	1	2	PB000111.01.0		d, 1, P	MAL12P1.63	
	tag 57	1	1	2	PB000120.01.0	*SIS22*	Δ, P	PFC1065w	
	tag 26	1	1	2	PB000133.00.0	*SIS23*	Δ	--	
	tag 59	0	2	2	PB000171.01.0	*SIS24*	Δ	--	
	tag 339	0	2	2	PB000178.02.0	*SIS25*	Δ, 1	PFE0810c	40S ribosomal subunit protein S14
	tag 139	0	2	2	PB000207.01.0	*SIS26*	Δ, 1	PFL1050w	SP, TRAF-like
	tag 221	0	2	2	PB000297.01.0	*SIS27*	Δ, 1	PF11_0251	SP, Endoplasmic reticulum oxidoreductin
	tag 12	0	2	2	PB000307.01.0	*UIS9-7*	a, 1	PFE0935c	RNA-binding protein
	tag 134	0	2	2	PB000352.00.0	*SIS28*	Δ, 1	PF11_0150	SP, 6TM, Rhomboid family, SecY protein
	tag 51	2	0	2	PB000352.02.0		d, 1, P	PFL2440w	DNA repair protein rhp16
	tag 317	1	1	2	PB000354.03.0		d, 1	PF10_0283	TM
	tag 285	0	2	2	PB000396.01.0	*SIS29*	Δ, 1	PFC0330w	PDZ domain protein
	tag 292	0	2	2	PB000412.02.0	*SIS30*	Δ, 1	PFI0750c	
	tag 17	0	2	2	PB000424.02.0		d, 1	PF11_0358	DNA-directed RNA polymerase, beta subunit
	tag 320	0	2	2	PB000450.03.0	*SIS31*	Δ, 1	MAL13P1.148	myosin
	tag 54	0	2	2	PB000530.00.0		d, 1	PFE0400w	ankyrin
	tag 277	0	2	2	PB000553.03.0		d, 1	PF13_0161	
	tag 303	1	1	2	PB000591.00.0	*SIS32*	ΔΔ	PFC0125w	DNAJ domain protein
	tag 184	0	2	2	PB000599.03.0	*SIS33*	Δ, 1	PF14_0681	Diacylglycerol kinase
	tag 34	0	2	2	PB000604.01.0		d	PFF0840w	
	tag 87	0	2	2	PB000610.02.0	*SIS34*	Δ, 1	PFL2390c	
	tag 204	0	2	2	PB000624.01.0	*SIS35*	Δ, 1	PF13_0261	ATP binding protein
	tag 128	0	2	2	PB000650.00.0	*SIS36*	Δ, 1, P	PF14_0446	Fasciclin domain
	tag 58	0	2	2	PB000675.02.0	*SIS37*	ΔΔ	PFF1450w	Sec14-like cytosolic factor
	tag 144	1	1	2	PB000709.00.0	*SIS38*	Δ, 1	PF14_0181	Calmodulin
	tag 289	2	0	2	PB000798.02.0	*MAEBL*	d, 1, P	PF11_0486	MAEBL, SP, TM
	tag 88	0	2	2	PB000807.01.0	*SIS39*	Δ, 1	PFD0485w	Ferlin like protein
	tag 353	0	2	2	PB000811.01.0		d, 1	MAL7P1.125	
	tag 80	1	1	2	PB000837.02.0	*SIS40*	Δ, 1	PF14_0695	DNA-directed RNA polymerase, alpha subunit
	tag 116	0	2	2	PB000857.01.0	*SIS41*	Δ, 1, P	PF11_0183	GTP-binding nuclear protein ran/tc4
	tag 238	0	2	2	PB000874.02.0	*SIS42*	Δ, 1	MAL8P1.142	Proteasome beta-subunit
	tag 79	0	2	2	PB000903.01.0	*SIS43*	Δ, 1	PF11_0197	SP, Ankyrin repeat
	tag 227	0	2	2	PB000904.01.0	*SIS44*	Δ, 1	PF11_0196	SP
	tag 206	0	2	2	PB000927.03.0	*SIS45*	Δ, 1	MAL12P1.113	Heat shock protein DNAJ homologue Pfj4
	tag 210	0	2	2	PB000941.01.0	*SIS46*	Δ, 1	PF10_0096	Octapeptide repeat
	tag 349	0	2	2	PB000994.02.0*		d, 1	PFL1690w	
	tag 218	0	2	2	PB001066.02.0		d, 1	PF10_0140	SPRY
	tag 228	0	2	2	PB001080.02.0	*SIS47*	Δ, 1	PFL0870w	SP, TM, TSP, thrombospondin-related 3
	tag 267	1	1	2	PB001118.02.0	*UIS4-6*	d, 1	PFA0170c	TM
	tag 27	0	2	2	PB001120.00.0	*SIS48*	Δ, 1	MAL13P1.150	2 TM
	tag 6	0	2	2	PB001130.00.0	*SIS49*	Δ, 1, P	MAL8P1.133	Glycosyltransferase family 28 protein
	tag 124	1	1	2	PB001157.02.0	*SIS50*	Δ, 1	MAL8P1.67	PIN domain like
	tag 301	0	2	2	PB001178.02.0	*SIS51*	Δ, 1	PFD0300w	
	tag 123	0	2	2	PB001253.00.0	*SIS52*	Δ, 1	PFD0360w	Zinc finger
	tag 331	0	2	2	PB001305.00.0	*SIS53*	Δ, 1, P	PF11_0168	Serine esterase
	tag 70	1	1	2	PB001374.02.0		d, 1, P	MAL13P1.304	Malaria antigen, metalloendopeptidase
	tag 31	0	2	2	PB001507.02.0	*SIS54*	ΔΔ	PFB0620w	
	tag 312	0	2	2	PB001517.02.0		d	PFF0800w	SP, TM, vWFA, TSP1
	tag 43	1	1	2	PB100341.00.0	*SIS55*	Δ	--	
	tag 93	1	1	2	PB101083.00.0		d	--	
	tag 61	2	0	2	PB101148.00.0	*SIS56*	Δ	--	
	tag 114	2	0	2	PB101408.00.0	*SIS57*	Δ, 1	PFE1260c	TM
	tag 56	2	0	2	PB101596.00.0	*SIS58*	Δ	--	
	tag 342	0	2	2	PB102211.00.0	*SIS59*	Δ	--	
	tag 318	1	1	2	PB102277.00.0		d	--	
	tag 71	0	2	2	PB103980.00.0	*SIS60*	Δ	--	2 TM
	tag 271	0	2	2	PB105487.00.0		d	--	2 TM
	tag 35	0	2	2	PB106484.00.0	*SIS61*	Δ, 1	PF14_0449	
	tag 208	0	2	2	PB108086.00.0	*SIS62*	Δ	--	
	tag 151	0	2	2	PB000618.02.0	*SIS63*	Δ	PFD0725c	arsenical pump-driving ATPase, putative
	tag 179	1	1	2	PB300567.00.0	*SIS64*	ΔΔ	PFL2420w	
	tag 85	0	2	2	PB300953.00.0	*SIS65*	ΔΔ	MAL8P1.121	
	tag 33	1	1	2	PB301273.00.0		d, 1	PFL1695c	
	tag 47	0	2	2	PB000460.01.0	*SIS66*	Δ, 1	PFC0750w	SP

(a) : mRNA detected in *P. berghei *sporozoites [14].

(d): mRNA detected in *P. yoelii *sporozoites [13].

Pb : detected by proteomics in *P. berghei *sporozoites [11].

P : detected by proteomics in *P. falciparum *sporozoites [10].

1 : detected in *P. falciparum *sporozoites with microarrays [9].

SP : signal peptide.

TM : transmembrane domain.

** : orthologue of a *P. yoelli *gene for which a *P. berghei *gene is not yet annotated.

SIS: new **S**porozoite expressed gene **I**dentified by **S**AGE, this work.

Δ : no rodent Plasmodium sporozoite ESTs or proteomics data.

ΔΔ : no sporozoite ESTs or detection by microarrays or proteomics in *Plasmodium *species.

### Validation of SAGE data

SAGE, like microarrays, is designed to give quantitative information on gene expression and the interpretation of the results depends on correct gene identification. This identification is not always straightforward in *P. berghei *since genomic sequence clusters are generally shorter than in *P. falciparum*; this results in the prediction of a large number of truncated genes, often lacking 5' or 3' ends. In addition, there are less EST ressources.

As a first step towards confirming the gene expression in sporozoites and validating tag annotation, we obtained more accurate data on gene structure and organization, by clustering ESTs and comparing with *P. yoelii *and *P. chabaudi *orthologous sequences. In Table 1, 15 genes (indicated by *) have been manually reannotated resulting in a different structure and a longer ORF than that predicted in the databases. These structures have been confirmed for 13 genes by RT-PCR on sporozoite RNA (see Additional file 2). For genes that are split between two genomic clusters, the intervening fragment was cloned and sequenced (see Additional file 2).

To confirm the identification of the 66 novel genes expressed in salivary gland sporozoites, RT-PCR experiments were performed. PCR fragments were obtained for all of them indicating that these genes are truely expressed (Additional file 3).

**Figure 2:**
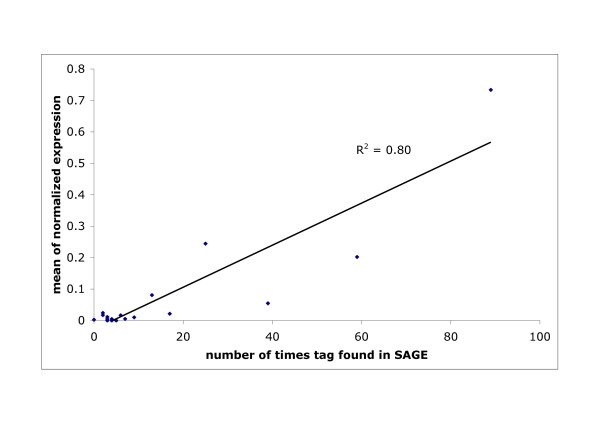
Correlation between the levels of gene expression as determined by qPCR and the number of times the identifying tag sequence was found in the SAGE library. R^2 ^is the coefficient of correlation.

The number of times a tag sequence is identified in a SAGE library is expected to correlate with the relative abundance of the steady state mRNA [17]. To determine whether the SAGE data correctly reflects transcript abundances, we selected eighteen known or novel genes, predicted to be expressed at high or low levels, and quantified their RNA by qPCR in sporozoites isolated from SG at d18 of infection. The values were normalised to the reference gene PB001026.00.0 (*CS*) and plotted against the number of times the identifying tag was found in the SAGE library at d18 of infection (Figure 2). A good correlation (R^2 ^= 0.8) was found and was even greater (R^2 ^= 0.96, not shown), when a second abundant tag sequence for two genes, *UIS4 *and *TRAP*, was taken into account. Thus, the number of times a tag was found in our SAGE data correctly reflects the gene expression levels in sporozoites.

### Hierarchical classification of sporozoite expressed genes

In Table 1, the 123 genes unambiguously identified by this SAGE as being expressed in SG sporozoites have been classified into three groups according to the number of times the identifying tag sequence was found in the combined d14 and d18 libraries (with the caveat that for some genes there may be an underestimation due to the criteria used above).

#### Group 1

The first group of highly expressed genes, defined by tags found more than 20 times in the combined libraries, contains five genes of which four have been described previously: PB000374.03.0, which codes for TRAP, a major sporozoite protein having an essential role in sporozoite motility [22]; PB100551.00.0 and PB107461.00.0, which code for UIS4 and UIS7 respectively, and were identified by SSH as genes whose expression is upregulated in SG sporozoites compared to midgut sporozoites [14]; PB001063.00.0, also known as *S23*, which codes for a reticulocyte binding protein and was identified by SSH as a gene upregulated in SG sporozoites compared to blood stages [15].

**Figure 3:**
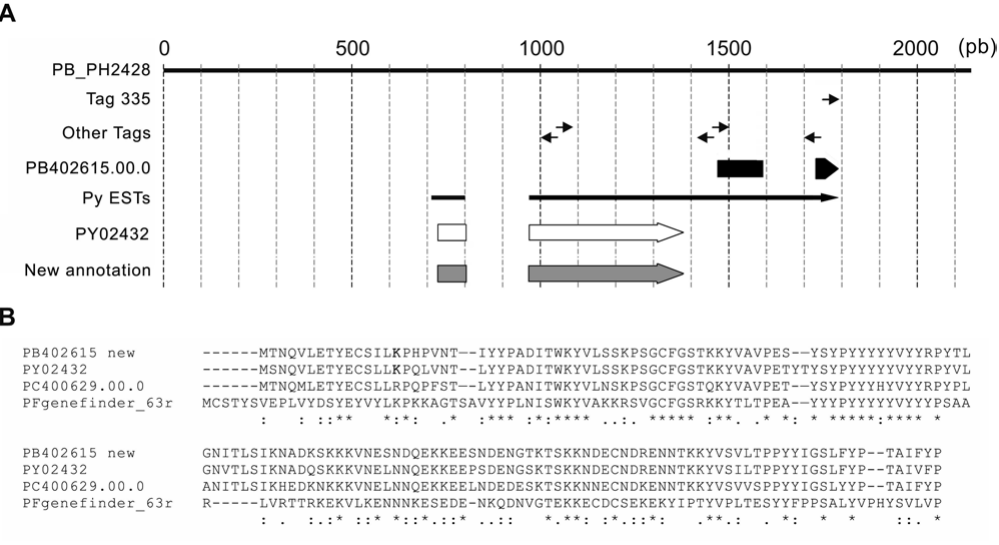
New annotation of PB402615.00.0. A) Continuous black line corresponds to the *P. berghei *genomic DNA contig; the position of tags are indicated by small arrows (→: sense tag); (←: antisense tag); thick black arrow: PB402615.00.0 as annotated in PlasmoDB; thin black arrow: EST cluster; thick white arrow: ORF of *P. yoelii *orthologue; thick grey arrow: new annotation of PB402615.00.0 ORF. B) Alignement of protein sequences of PB402615.00.0 orthologues in *P. yoelii*, *P. chabaudi *and *P. falciparum*.

Surprisingly, the gene PB402615.00.0, for which the identifying tag (tag 335) was found the most often, has not been described previously. The gene sequence aligns with numerous ESTs of *P. yoelii *sporozoites and parasites developing in the absence of host cells [13,23]. To further characterize the gene structure, a manual clustering of these ESTs was performed. Several tags were found along this cluster, both in sense and antisense orientations and, as expected, the most 3' tag was also the most abundant (Figure 3A). The other internal tags, one of which is highly represented (tag 189, see additional file 1), may be due to either alternative polyadenylation events or priming at internal polyA stretches during cDNA synthesis, as hypothesized by others [24]. To rule out the possibility that the cluster covers two different genes, a RT-PCR was performed using primers at the 5' and 3' end of the cluster and this detected a unique transcript of the predicted size (data not shown). Based on our results and annotation, we propose that the ORF predicted for PB402615.00.0 during automatic annotation is incorrect and that this gene codes for a 17 KDa protein, rich in tyrosine and basic amino acids. Furthermore, this new annotation indicates that there are orthologous sequences in *P. yoelii *(PY02432), *P. chabaudi *(PC400629.00.0) and also in *P. falciparum *(PFgenefinder_63r.) (Figure 3B).

Interestingly, PB402615.00.0 is not the only gene characterized by several abundant tags. For instance, two tags are characteristic of PB100551.00.0, encoding UIS4: the most abundant (tag 186, found 95 times) is located at the end of the ORF, while the other (tag 153, found 74 times), is located 650 nt downstream and probably defines a transcript with a longer 3' UTR. Such observations may be useful when defining the gene structure with the complete potential regulatory sequences.

#### Group 2

The second group contains eight moderately expressed genes defined by tags found 10 to 20 times. Among these, there is one *UIS *gene (*UIS10*/PB000484.00.0) and three *S *genes *S13*/*SPECT2 *(PB000252.01.0), *S21/PbTRSP *(PB000881.01.0) and *S12 *(PB104017.00.0). *UIS10/PbPL *codes for a lecithine-cholesterol acyl-transferase involved in cell traversal [25]. The S13/SPECT2 protein is characterized by a MAC/Perforin domain and was found to be essential for membrane attack during cell invasion [26]. The protein S21/PbTRSP, which contains a thrombospondin type 1 domain, has recently been shown to have a role in host cell invasion [27]. Finally, *S12 *codes for a protein with a predicted signal peptide, but whose function has yet to be characterized.

The gene PB000649.01.0 codes for the cysteine protease ECP1, involved in egress of sporozoites from oocysts [28]. Two genes, PB000891.00.0 (*Pbs36p*) and PB000400.01.0, encode potential GPI- anchored proteins of the Pfs48/45 family, orthologues of *P. falciparum *Pf52 and Pfs38 proteins [29]. Proteins of this family, characterized by a 6-Cys domain, are involved in intra-hepatic development and/or gametocyte fertilization [21,30,31]. Pfs38 expression was previously observed in *P. falciparum *sporozoites [9] but not in rodent sporozoites. We have named PB000400.01.0, *SIS1*, for Sporozoite expressed gene Identified by SAGE1 (see Table 1). Finally, PB000317.00.0, whose structure has been manually revised, codes for a potential secreted and GPI-anchored protein and therefore constitutes a novel candidate that could be involved in host cell recognition/invasion.

#### Group 3

Of the remaining 110 genes, expressed at low levels and defined by tags found less than ten times, 43 aligned with *P. yoelii *or *P. berghei *sporozoite ESTs, one was detected in *P. berghei *sporozoites by proteomics and PB000892.00.0/*Pbs36 *has been described in sporozoites [21]. Thus 65 genes in this group are shown for the first time to be expressed in this stage of the *P. berghei *parasite (indicated as *SIS2-66 *on Table 1). Orthologous *P. falciparum *genes are predicted for 90 genes in this group, and expression in sporozoites has been detected for 74 by microarrays (of which 20 also by proteomics) and one by proteomics only. The 20 *P. berghei *predicted genes for which there are no obvious *P. falciparum *orthologues could correspond to incomplete genes and require more precise annotation. Alternatively, they may be highly divergent genes or genes whose prediction has been missed during *P. falciparum *genome annotation.

Nine genes in this group have been described previously: AB125370 (*SPECT*) and PB000892.00.0 (encoding Pbs36) are important for liver infectivity [21,32] and PB000798.02.0, encoding MAEBL, has a role in the initial binding of sporozoites to the mosquito SG [33]. Six genes, *UIS1*, *UIS2*, *UIS9-7/UIS29-1*, *UIS4-6*, *S25*, *S14*, were already identified as being expressed in SG sporozoites [14,15].

In addition, two other genes could have a potential role in adhesion/invasion of host cells: PB001517.02.0 codes for a protein with a Thrombospondin 1 domain and a vonWillebrand type A domain, and PB001080.02.0, which encodes a protein defined as thrombospondin-related 3 (also found as AF375983 in Genbank). Proteins involved in the molecular motor needed for sporozoite motility are also represented by aldolase (PB000757.02.0), which provides the link between TRAP and myosinA [34,35], and by a kinesin-related protein (PB000464.00.0/*S25*). While myosinA, which is thought to play a major role in the motility of Apicomplexa zoites, is absent from our description (with a tag found only once), another gene encoding a potential myosin is identified (PB000450.03.0). Two genes encoding proteases of the rhomboid family (PB001432.02.0/*SIS14 *and PB000352.00.0/*SIS28*) are also found. Interestingly, these proteins are the orthologues of PfROM4 and PfROM1, respectively, which are able to cleave adhesins, such as TRAP, AMA1, MAEBL and others, that are involved in interactions with host-cell receptors [36].

The expression of only two genes encoding ribosomal proteins has been detected, confirming, as previously reported [13], their under-representation in the sporozoite transcriptome. Interestingly, two genes coding for proteins with RNA-binding domains are identified, one of which has a pumilio domain suggesting a role in the negative regulation of translation [37]. These proteins were previously described as being upregulated in *P. falciparum *gametocytes and sporozoites [9] and may be involved in the regulation of sporozoite protein expression.

Other genes found in this group code for proteins with diverse biological functions for example energy metabolism, signal transduction and protein modification. Interestingly, a gene encoding a putative sugar transporter (PB000161.03.0), whose *P. falciparum *orthologue is expressed specifically in sporozoites, is present [9]. We previously reported an increase in mRNA levels of an *Anopheles *sugar transporter in infected SG [16]. These two transporters may play a role in providing the sporozoite with sufficient energy resources for its journey from the oocysts to the SG and from the bite site to the liver in the vertebrate host. The presence of three genes coding for proteins involved in iron-sulphur cluster formation and iron homeostasis can also be noted. Iron-sulphur cluster formation is essential for a wide variety of processes, including facilitation of electron transfer in oxidative phosphorylation and enzymatic activities in mitochondria, cytoplasm or nucleus as well as sensing of intracellular iron and/or oxidant levels. Expression of these genes may be preparing the sporozoite for high mitochondrial activities related to motility and/or for the future iron-rich blood environment.

### Expression profiles of sporozoite expressed genes

**Figure 4:**
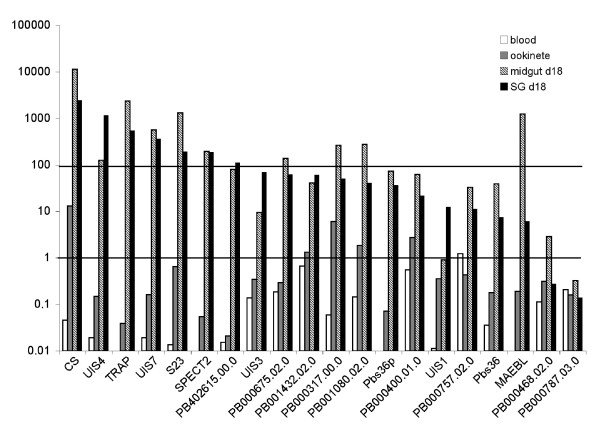
Histogram representation of qPCR analysis of gene expression in mixed blood stages, ookinetes, d18 midgut sporozoites and d18 salivary gland sporozoites. X axis: genes tested; Y axis: log scale of mean normalised expression.

To further characterize a number of genes that were not previously studied, namely PB402615.00.0 (group1), PB000317.00.0 and PB000400.01.0 (group2), PB001080.02.0 (Thrombospondin-related 3 protein), PB000757.02.0 (aldolase), PB001432.02.0 (rhomboid protein), PB000787.03.0 (arginase), PB000468.02.0 (eukaryotic translation initiation factor 5) and PB000675.02.0 (sec14-like protein) (group 3), we compared their expression profiles with those of known transcripts such as *CS*, *UIS1*, *UIS3*, *UIS4*, *UIS7*, *TRAP*, *S23*, *Pbs36, Pbs36p*, *MAEBL *and *SPECT2*. The RNA levels were quantified by qPCR in ookinetes, midgut and SG sporozoites and blood stages and the results were normalised with respect to *hsp70 *which is expressed at all stages (Figure 4).

All genes showed higher levels of expression in midgut and/or SG sporozoites compared to ookinete or blood stages, with the exception of PB000787.03.0 whose expression is clearly not sporozoite specific. Other genes, for example PB000400.01.0, PB000757.02.0 and PB001432.02.0 are easily detectable in blood stages, suggesting they may also have a role at this stage. Finally, PB001432.02.0, PB000317.00.0, PB001080.02.0 and PB000400.01.0 are expressed, like *CS*, at relatively high levels in the ookinete stage, although this does not mean that the protein is produced.

As observed in Table 1, the number of times a tag is found in the SG libraries at d18 of infection often differs to that at d14. This is due in part to the higher number of tags sequenced in the d18 library (2 fold) and to the increase (2 fold) in the number of sporozoites inside the glands at d18. However, the tags of some genes, for example *UIS4*, increase up to 15 fold between d14 and d18 whereas they decrease for others. This suggested that there might be variations in gene expression during sporozoite storage in the glands. To further characterize these variations, the relative levels of gene expression for the same panel of genes as above were determined in sporozoites isolated from SG and midguts of *A. gambiae *mosquitoes at d14 or d18 of infection. The values were calculated from the geometric averages and normalised to the geometric mean of PB001026.00.0 (*CS*), as this gene was determined to be the best reference using GeNORM software.

The results show important increases (4–66 times) in RNA amounts in SG compared to midgut sporozoites for *UIS4, UIS1, UIS3, SPECT2*, *UIS7 *and PB402615.00.0 at d18 (see Table 2). These results were expected for the *UIS *genes as they were found in an SSH library between SG and midgut sporozoites [14]. They also suggest that PB402615.00.0 may be important for preparing the sporozoites for infection. On the contrary, there is significantly less *MAEBL *RNA in SG compared to midgut sporozoites in agreement with MAEBL having a role in sporozoite adhesion to mosquito salivary glands [33].

**Table 2:** Variation of gene expression in *P. berghei *sporozoites developping in *A. gambiae*.

		**between tissues**		**with time**	
			
**Gene ID**	**Gene name**	**ratios at d14 SG/midgut**	**ratios at d18 SG/midgut**	**ratio SG d18/d14**	**ratio midgut d18/d14**
PB100551.00.0	*UIS4*	37.07*	66.31**	4.17*	2.33*
PB108190.00.0	*UIS1*	20.72*	56.52**	2.15*	0.79*
PB000892.03.0	*UIS3*	20.88*	54.45**	3.77*	1.45*
PB402615.00.0		8.22*	10.67**	1.57*	1.21*
PB000252.01.0	*SPECT2*	7.55*	7.69**	1.24*	1.22*
PB107461.00.0	*UIS7*	5.81*	4.59**	2.55*	3.24*
PB000891.00.0	*Pbs36p*	4.67*	3.79**	0.77*	0.95*
PB000787.03.0		1.13*	2.32**	1.54*	0.75*
PB000675.02.0		2.50*	2.13**	0.76*	0.90*
PB000400.01.0		7.52*	1.97**	0.49*	1.88*
PB000892.00.0	*Pbs36*	1.54*	1.89**	1.28*	1.04*
PB000374.03.0	*TRAP*	1.57*	1.88**	1.22*	1.01*
PB000757.02.0		1.71*	1.64**	0.71*	0.74*
PB001432.02.0		1.54*	1.35**	0.75*	0.86*
PB001063.00.0	*S23*	1.76*	1.00**	0.80*	1.41*
PB000317.00.0		1.12*	0.87**	0.77*	0.99*
PB001080.02.0		1.20*	0.70**	0.75*	1.29*
PB000468.02.0		0.52*	0.44**	0.90*	1.05*
PB000798.02.0	*MAEBL*	0.04*	0.04**	0.87*	0.87*

* :p = 0.05 ** :p = 0.01

In addition, the ratios obtained between d18 and d14 SG sporozoites indicate that there is a significant increase in expression over this period for *UIS4, UIS1, UIS3 *and *UIS7*, whereas there is no substantial change in RNA quantities for the other genes. Similar increases in expression were also seen for *UIS3 *and *UIS4 *in *A. stephensi *infected salivary glands suggesting that *P. berghei *sporozoites develop similarly in *A. gambiae *and *A. stephensi *(not shown).

Taken together, the qPCR data show that sporozoites are capable of modulating their RNA amounts between the midgut and salivary glands, as well as during their storage within the latter.

## Discussion

Although the transcriptional repertoire of *Plasmodium *sporozoites has been investigated in several laboratories using different techniques, including cDNA libraries, SSH, microarrays and proteomics, only the microarray data has provided information concerning the level of gene expression [9]. We have obtained new data concerning the transcriptional repertoire of *P. berghei *sporozoites using SAGE on *A. gambiae *infected salivary glands. SAGE does not require *a priori *knowledge of the sequence of genes to be analysed and provides quantification of gene expression by the number of times a tag sequence is obtained [17]. However, since the sequence and annotation of the *P. berghei *genome is incomplete, the attribution of a tag sequence to a gene was not straightforward.

Several criteria were applied before retaining a tag sequence as a gene identifier: it was found twice or more in the cumulative SAGE libraries, the BLASTN analysis gave a unique hit in the genome, which was in the sense orientation of an annotated gene and was located at the most 3' predicted *Nla*III site or within 500 nt of the stop codon of a neighbouring gene. These combined criteria resulted in the unambiguous identification of 123 genes expressed in SG sporozoites, of which 16 were already known to be sporozoite-expressed genes: *TRAP*, several *UIS *and *S *genes, *SPECT*, *Pbs36p*, *Pbs36 *and *MAEBL*. It should be noted that this list of 123 *P. berghei *SG sporozoite-expressed genes is not exhaustive.

Of the 2154 unique tag sequences found at least twice in the libraries from infected mosquitoes, only 25% (530) matched perfectly to the present version of the *P. berghei *genome. The 1624 remaining tags may derive from *A. gambiae *cDNAs. Indeed, 587 match on *A. gambiae *ESTs and 943 on the *A. gambiae *genome (see Additional file 1, sheet 2). Alternatively, they may derive from genes not yet sequenced in the *P. berghei *genome. In addition, the absence of matches of some tags could be due to polymorphisms between the *P. berghei *strain sequenced, ANKA, and the NK65 strain used in our SAGE.

Also, 202 (55%) of the tag sequences that gave a single hit in the *P. berghei *genome were not found within an annotated gene. Upon more detailed analysis of their position in the contigs, 63 were found to be within 500 nt of the stop codon of an annotated gene, in the sense orientation and were considered to derive from transcripts of these genes. However, we were unable to place 139 tag sequences either within an annotated ORF or within 500 nt of a stop codon. These tags should help in refining the present annotation of the *P. berghei *genome and may, in the future, be formally proven as corresponding to sporozoite-expressed genes.

Among the 368 tag sequences that gave a single match in the *P. berghei *genome, 64 were found in the antisense orientation, either within annotated genes (56) or within 500 nt of the stop codon of an annotated gene (8). Antisense RNAs have been described previously in *P. falciparum *and they are suggested to be involved in transcriptional regulation [38-40]. At the present time, there is insufficient *P. berghei *cDNA data and no microarrays using sense and antisense probes, to establish whether or not these tags correspond to antisense RNAs or to transcripts from a gene on the opposite strand.

The stringent criteria described above resulted in the elimination of the vast majority of tag sequences, retaining 0.7% (123/17181) of the unique tag sequences found only in the infected libraries, representing 3% (668/21721) abundance. Among the tag sequences rejected there are a number that matched with genes known to be expressed in sporozoites, such as *CS*, *UIS3*, *RON4 *and *AMA-1*. Consequently, other genes expressed in sporozoites may have been eliminated. Furthermore, due to the SAGE cloning procedure, genes for which the transcripts have no *Nla*III site will also be missing. The data supplied in the supplemental tables should be useful to other researchers interested in genes expressed in sporozoites.

Amongst the *P. berghei SIS *genes identified in this study as being expressed in sporozoites, eighteen are predicted to encode proteins with one or more transmembrane regions and/or a signal peptide sequence, suggesting that they are membrane associated or secreted proteins. Other genes of potential interest are PB000464.00.0/*S25*, a kinesin-related protein, which could be involved in motility; PB000416.02.0, a putative RNA-binding protein of the pumilio/mpt5 family known for their role in repression of gene expression; PB000903.01.0/*SIS43 *which contains an ankyrin repeat suggesting a role in protein-protein interaction; PB000650.00.0/*SIS36*, which contains a fasciclin domain, may have a role in cell adhesion. Finally, PB402615.00.0, for which the identifying tag was the most abundant in this SAGE analysis, is annotated as a hypothetical protein and aligns with *P. yoelii *ESTs from sporozoites as well as parasites developing in the absence of host cells. Our qPCR data show that this gene is expressed in sporozoites but not in ookinetes or blood stages and that there is a substantial increase in the amount of RNA for this gene between midgut and salivary gland sporozoites. This differential regulation between organs and the mRNA abundance in SG suggest a role for this gene in sporozoite infectivity in the mammalian host.

Several properties (motility, infectivity, etc) differ between midgut and SG sporozoites, but it is not known whether these developmental changes are time and/or tissue dependent nor which signalling factors are involved. Interestingly, the qPCR data presented here indicate that, at least for *UIS3 *and *UIS4*, not only the tissue (midgut versus salivary gland) but also the time spent in the SG significantly influences the level of expression of individual genes. This change in expression of genes that are essential for development in the liver, is in agreement with the increase in infectivity of SG sporozoites between d14 and d18 post blood meal [41].

Since SAGE provides a quantitative read out of gene expression, and as our qPCR analysis confirmed this, the 123 genes were classified into 3 groups according to the number of times the tag was found in the libraries. Among the thirteen genes presenting the most abundant tags (groups 1 and 2), there are seven, *UIS4*, *TRAP*, *S13/SPECT2*, *S21/Pb TRSP*, *ECP1*, *UIS10/Pb PL*, and *Pbs36p*, that have been shown via gene knockout experiments to be essential for the sporozoite or liver stage development [7,21,22,25-28]. Furthermore, among the genes with less abundant tags there are three, *SPECT*, *Pbs36 *and *MAEBL*, which have also been shown to have essential roles [21,32,33]. This indicates that our approach has identified several genes known to be essential for sporozoites and points to additional genes that may be required at this stage. It will be of interest to inactivate the novel sporozoite-expressed genes identified in this paper to define their function in the parasite. Mutants that are defective in their development in the mammalian host would be of particular interest as they could provide new tools to probe the host immune response to *Plasmodium *infection.

## Conclusion

The SAGE described here has lead to the identification of 66 novel genes expressed in *P. berghei *sporozoites. These novel sporozoite expressed genes, especially those expressed at high levels in salivary gland sporozoites, are likely to play a role in *Plasmodium *infectivity in the mammalian host.

## Methods

### Mosquito Infections

*A. gambiae *(Yaounde strain) and *Anopheles stephensi *(*A. stephensi*) (Sda500 strain) mosquitoes were reared at the Centre for Production and Infection of Anopheles at the Pasteur Institute under standard conditions [42]. 3–4 day old female mosquitoes were fed on anaesthetised *P. berghei *infected mice. A recombinant *P. berghei *strain expressing GFP in late midgut stages and SG of mosquitoes was used [43].

### Construction of SAGE libraries

The construction of the SAGE libraries has been described elsewhere [16]. Briefly, 2 libraries were constructed from SG of infected *A. gambiae *mosquitoes: one 14 days (early stage of infection) and the other 18 days (stage at which sporozoites are considered to be fully infectious) after feeding on *P. berghei *infected mice. Two control libraries were made from SG of mosquitoes 14 and 15–18 days post-feeding on uninfected mice.

### Analysis of Tag Sequences

The analysis of tag sequences has been described in detail in [16]. Tag sequences found only in libraries from the infected mosquitoes were considered as potential *P. berghei *sequences. BLASTN comparisons were performed against the library of *P. berghei *contigs and annotated genes obtained from the ftp site of the Sanger Institute *P. berghei *genome project (version 12 August 2005). Sequences of genes unambiguously identified by tags were aligned to EST_others (i. e. non-human, non-mouse EST sequences) at the National Center for Biotechnology Information via the *Plasmodium *genome consortium PlasmoDB.

### Real-time Quantitative RT-PCR

Real time qPCR was performed on cDNA preparations using the SYBR green detection system and the ABI Prism 7900 sequence detector (Applied Biosystems) according to manufacturer's instructions. Midgut and SG sporozoites were isolated from *A. gambiae *mosquitoes infected with *P. berghei *at d14 and d18 after the blood meal. Under these conditions, the mean number of sporozoites isolated from midguts was 5500 ± 200 (d14) and 7500 ± 1700 (d18), and the mean number of sporozoites from SG was 1500 ± 20 (d14) and 3900 ± 1200 (d18). SG sporozoites were also isolated from *A. stephensi *mosquitoes infected with *P. berghei *with slightly higher mean numbers of sporozoites per gland: 3300 ± 250 on d14 and 17000 ± 1800 on d18. RNA was extracted with TriReagent, DNAse treated, and cDNAs synthesised with Superscript II reverse transcriptase (Invitrogen) using random primers. Three independent RNA preparations were made for each sample. cDNAS of *P. berghei *ookinetes and asynchronous blood stages were prepared as described in [44]. PCR conditions were 1 cycle of 50°C for 2 min, followed by 95°C for 10 min, then 40 cycles of (95°C 15 sec, 60°C 1 min). qPCR was performed in triplicate. The standard curve was analysed for all primers and gave amplification efficiencies of 90–100%. Data was analysed with SDS 2.1 software and with geNORM Visual Basic application for Microsoft_Excel as described by Vandesompele *et al*. to determine the best reference gene for the qPCR [45]. This approach relies on the principle that the expression of a perfect reference gene should be identical in all samples, independent of experimental conditions or cell type. For the comparaison between midgut and salivary gland samples the Circumsporozoite gene (*CS*), PB001026.00.0, was determined to be the best reference gene and the expression of genes of interest were subsequently normalized to the geometric mean of this reference. The normalized gene expression was then calculated with the Q-Gene software [46]. For the qPCR analysis between different tissues the gene coding for hsp70 was found to be the best reference. The list of primers used for qPCR analysis is provided in Additional file 4.

### RT-PCR

cDNA samples were prepared as for qPCR except that half of the mRNA was not treated with reverse transcriptase. PCR was performed with 1 unit of Platinum Taq polymerase, in manufacturer's buffer (InVitrogen) and 1.5 mM MgCl_2_. PCR conditions were : 1 cycle at 94°C for 2 min, 40 cycles of (94°C for 20 sec, 55°C or 52°C for 35 sec, 68°C for 1 min) and 1 cycle at 68°C for 10 min. Primers used for RT-PCR are shown in Additional file 4.

## Abbreviations

SAGE: Serial Analysis of Gene Expression

SG: salivary glands

cDNA: complementary DNA

ESTs: expressed sequence tags

BLAST: Basic Local Alignment Search Tool

ORF: open reading frame

qPCR: quantitative polymerase chain reaction

KDa: kilodaltons

## Authors' contributions

IR-C was responsible for the conception of the SAGE, performed experiments, analysed data and wrote the manuscript.

TC, BB, SP and EB performed experiments, acquired and interpreted data and provided tables, figures and revised the manuscript.

JB and PC constructed the SAGE libraries and revised the manuscript.

RM and PBrey critically revised the manuscript

PBaldacci analysed the data and wrote the paper.

All authors read and approved the final manuscript.

## Supplementary Material

Additional file 1 List and annotation of the 530 tag sequences with hits in the *P. berghei *genome (sheet 1). BLASTN analysis against *A. gambiae *genome and EST sequences of the tags found specifically in infected salivary gland libraries that did not align with *P. berghei *genome (sheet 2). Click here for file

Additional file 2 Compilation of information in databases with BLASTN alignments and annotation of the 123 *P. berghei *genes with their putative fonction, predicted molecular weight and domains, expression data and *P. falciparum *orthologues. Click here for file

Additional file 3 Confirmation by RT-PCR of the expression of 66 *SIS *genes in salivary gland sporozoites. The SIS genes tested are indicated above the wells on cDNA samples (+RT) and controls for DNA contamination (-RT). The size marker is 100 nt Smart ladder (Eurogentec). G refers to gel number as indicated in Additional file 4. Click here for file

Additional file 4 Primers used for annotation and qPCR. Sheet1: List of primers used for annotation and results of RT-PCR analysis. Some PCR fragments have been sequenced and their sequences submitted to EMBL database (accession numbers are indicated in columns L and M). Column "E" refers to the gel in Additional file 3. Click here for file
