# Supplementary material for: Serial Analysis of Gene Expression in Plasmodium berghei salivary gland sporozoites
Source: BMC Genomics. 2007 Dec 19;8:466. doi: 10.1186/1471-2164-8-466 (PMC2263065; doi:10.1186/1471-2164-8-466)
Supplement: Additional file 3 — Confirmation by RT-PCR of the expression of 66 SIS genes in salivary gland sporozoites. The SIS genes tested are indicated above the wells on cDNA samples (+RT) and controls for DNA contamination (-RT). The size marker is 100 nt Smart ladder (Eurogentec). G refers to gel number as indicated in Additional file 4. [file 1471-2164-8-466-S3.PDF]

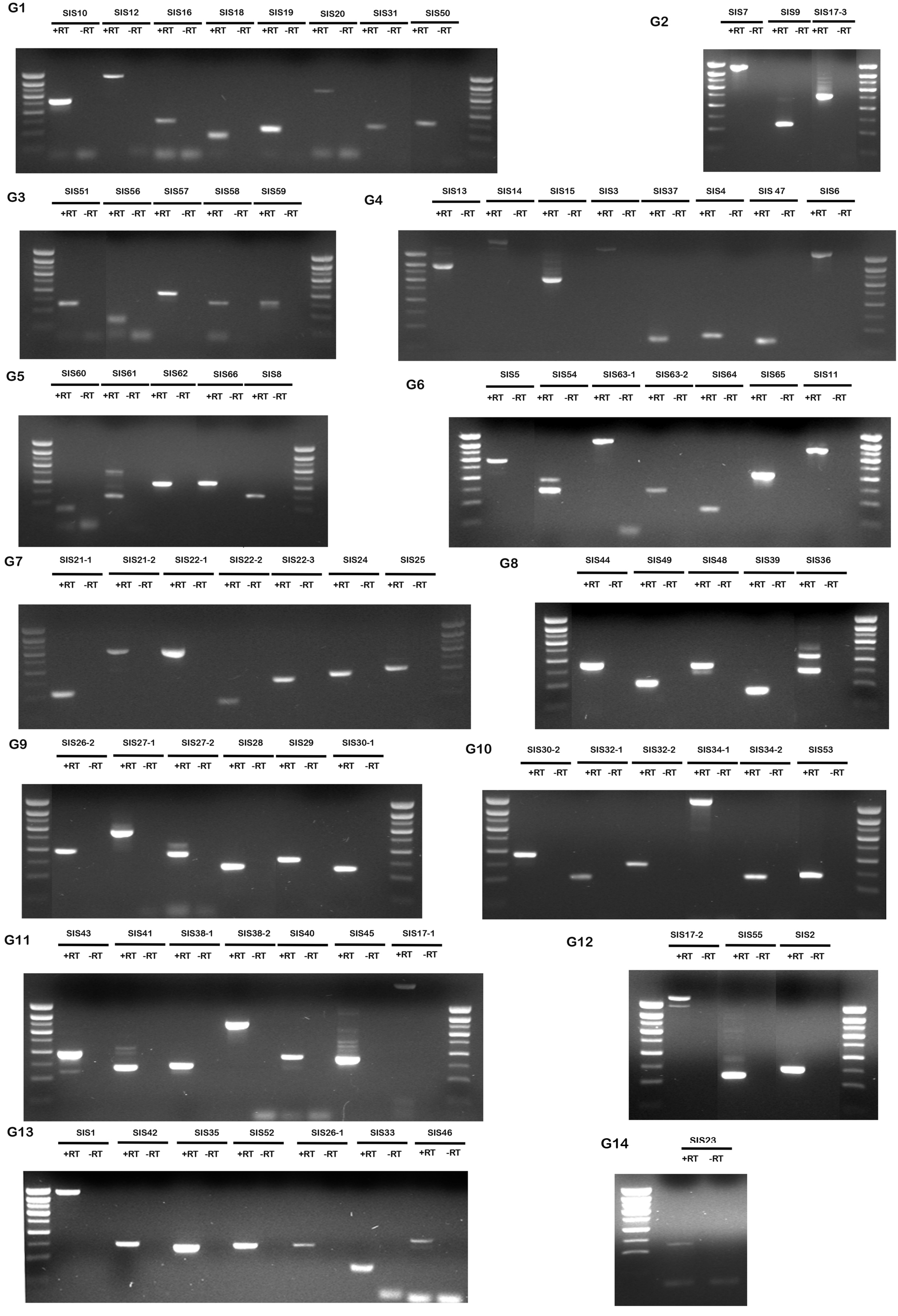
G1
SIS10 SIS12 SIS16 SIS18 SIS19 SIS20 SIS31 SIS50
+RT -RT
G2
SIS7 SIS9 SIS17-3
+RT -RT
G3
SIS51 SIS56 SIS57 SIS58 SIS59
+RT -RT
G4
SIS13 SIS14 SIS15 SIS3 SIS37 SIS4 SIS 47 SIS6
+RT -RT
G5
SIS60 SIS61 SIS62 SIS66 SIS8
+RT -RT
G6
SIS5 SIS54 SIS63-1 SIS63-2 SIS64 SIS65 SIS11
+RT -RT
G7
SIS21-1 SIS21-2 SIS22-1 SIS22-2 SIS22-3 SIS24 SIS25
+RT -RT
G8
SIS44 SIS49 SIS48 SIS39 SIS36
+RT -RT
G9
SIS26-2 SIS27-1 SIS27-2 SIS28 SIS29 SIS30-1
+RT -RT
G10
SIS30-2 SIS32-1 SIS32-2 SIS34-1 SIS34-2 SIS53
+RT -RT
G11
SIS43 SIS41 SIS38-1 SIS38-2 SIS40 SIS45 SIS17-1
+RT -RT
G12
SIS17-2 SIS55 SIS2
+RT -RT
G13
SIS1 SIS42 SIS35 SIS52 SIS26-1 SIS33 SIS46
+RT -RT
G14
SIS23
+RT -RT
